# Association between bone mineral density and scoliosis: a two-sample mendelian randomization study in european populations

**DOI:** 10.1186/s41065-024-00352-w

**Published:** 2024-12-31

**Authors:** Fangjun Yang, Jiantao Wen

**Affiliations:** 1 Department of orthopedic, Gansu Provincial Hospital of Traditional Chinese Medicine, Lanzhou, China; 2 Department of Pediatric Spine Surgery, Gansu Provincial Hospital of Traditional Chinese Medicine, Lanzhou, China

**Keywords:** Mendelian randomization, Scoliosis, Bone mineral density, Causality

## Abstract

**Background:**

Previous studies have shown that bone mineral density (BMD) has a certain impact on scoliosis. However, up to now, there is no clear evidence that there is a causal association between the two. The aim of this study is to investigate whether there is a causal association between BMD at different body positions and scoliosis by two-sample Mendelian randomization (MR).

**Methods:**

Genetic variants (SNPS) strongly associated with BMD (total body BMD (TB-BMD), lumbar spine BMD (LS-BMD), femoral neck BMD (FN-BMD), heel BMD (HE-BMD), and forearm BMD (FA-BMD)) were extracted from GEFOS and genome-wide association analysis (GWAS) databases SNPs) were used as instrumental variables (IVs). Scoliosis was also selected from the Finnish database as the outcome. Inverse variance weighting (IVW) method was used as the main analysis method, and multiple sensitivity analysis was performed by combining weighted median, MR-Egger, MR Multi-effect residuals and outliers.

**Results:**

IVW results showed that TB-BMD (OR = 0.83, 95%CI: 0.66–1.55 *P* = 0.13), LS-BMD (OR = 0.72, 95%CI: 0.52–0.99, *P* = 0.04), FN-BMD (OR = 0.74, 95%CI: 0.50–1.09, *P* = 0.13), FA-BMD (OR = 0.95,95%CI: 0.70–1.28, *P* = 0.75), HE-BMD (OR = 0.91, 95%CI: 0.77–1.08, *P* = 0.29). Sensitivity analyses showed no evidence of pleiotropy or heterogeneity (*p* > 0.05) (MR-PRESSO and Cochrane). The results were further validated by leave-one-out test and MR-Egger intercept, which confirmed the robustness of the study results.

**Conclusion:**

In conclusion, the present study demonstrates that the causal role of genetic prediction of scoliosis increases with decreasing lumbar BMD. There was no evidence that BMD at the remaining sites has a significant causal effect on scoliosis. Our results suggest that the lumbar spine BMD should be routinely measured in the population at high risk of scoliosis. If osteoporosis occurs, appropriate treatment should be given to reduce the incidence of scoliosis.

**Clinical trial number:**

Not applicable

**Supplementary Information:**

The online version contains supplementary material available at 10.1186/s41065-024-00352-w.

## Introduction

Scoliosis is a complex three dimensional (3D) structural deformity characterized by more than 10° of scoliosis on coronal radiographs of the spine with axial rotation and sagittal deviation [1]. According to the presence or absence of a clear cause, it can be divided into idiopathic and non-idiopathic [2, 3]. The global prevalence ranges from 0.47–5.2% [4, 5]. Despite extensive experimental and clinical studies over the years, its etiology and pathogenesis remain poorly understood. The etiology theory involves genetics, biomechanics, nervous system, endocrine system, spinal cord growth and bone metabolism [6]. Previous studies [7–11] have suggested that decreased bone mineral density (BMD) and even osteoporosis play an important role in the pathogenesis of scoliosis, but these studies have not suggested a potential cause-and-effect relationship.

To determine the causal relationship between BMD and scoliosis, we conducted a two-sample Mendelian randomization (MR) Study using public genome-wide Association Study (GWAS) data to explore the association. Mendelian randomization (MR) studies refer to the use of genetic variants (single nucleotide polymorphisms, SNPs) as instrumental variables (IVs) to infer causal associations of exposure levels (such as biomarkers) with outcomes [12]. Because SNPS are randomly assigned at conception (according to Mendelian’s second law), environmental confounders and disease states that develop later in life do not affect germline genetic susceptibility. Therefore, MR Limits bias due to confounding and reverse causality and allows causal inference [13, 14].

## Methods

### Study design

**Fig. 1:**
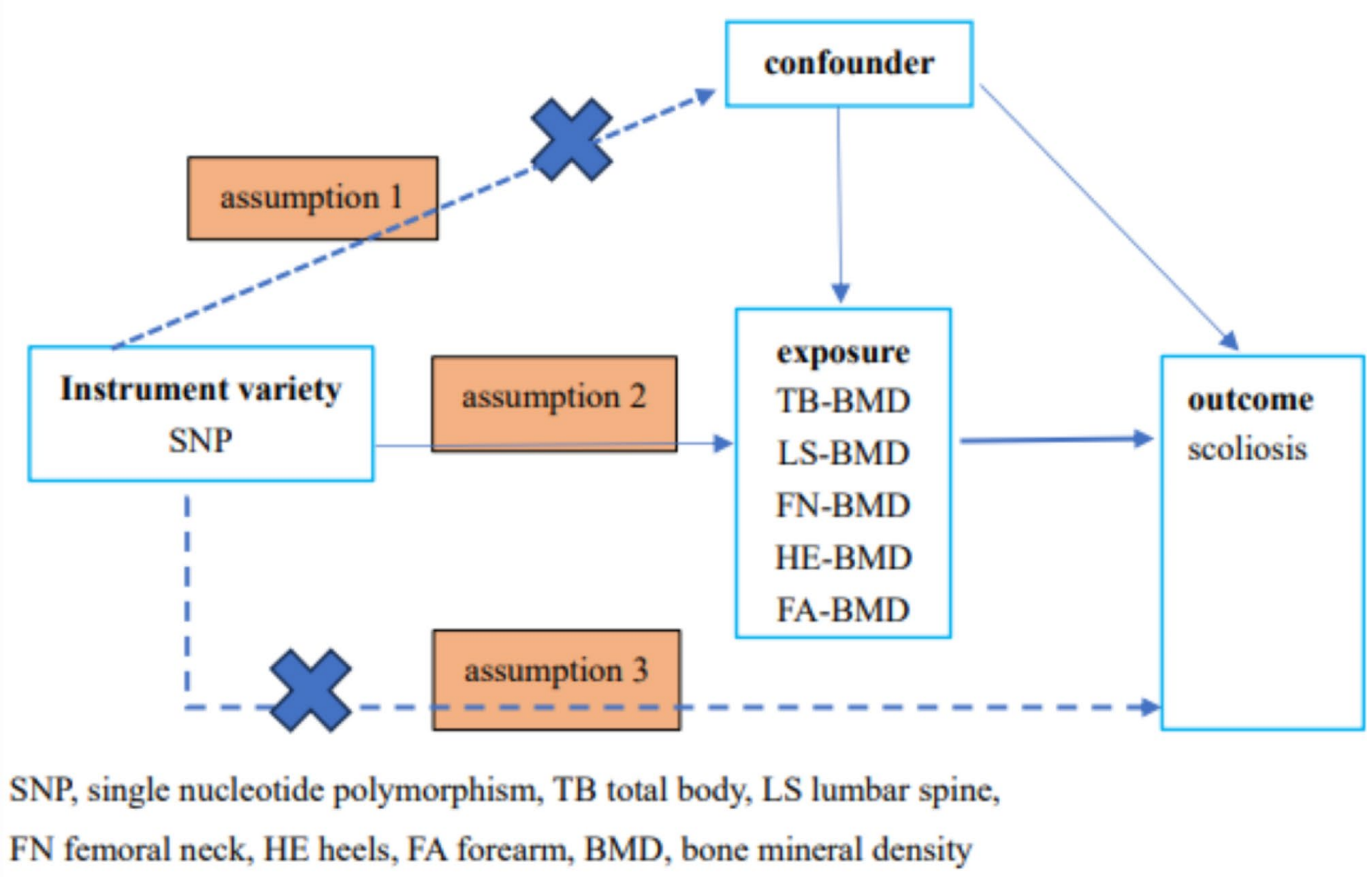
Schematic diagram of Mendelian randomization study

The flow of the study design is illustrated in Fig. 1. The IVs required for the MR Analysis must satisfy the following three assumptions [15–17]. (1) The IVs used were strongly associated with exposure (BMD at different sites); (2) The selected IVs were not related to potential confounders; (3) IVs could affect the risk of outcome (scoliosis) only through exposure.

### Data sources

Exposure data included total body BMD (TB-BMD), lumbar spine BMD (LS-BMD), femoral neck BMD (FN-BMD), forearm BMD (FA-BMD), and heel BMD (HE-BMD). Above all data from osteoporosis GEFOS genetic factors alliance (http://www.gefos.org/) and IEU Open GWAS (https://gwas.mrcieu.ac.uk/datasets/) summary statistics.

Scoliosis genetic statistics data from 2021 released R5 FinnGen alliance (https://r5.risteys.finngen.fi/). The large GWAS in Finns contained 1168 cases and 164,682 controls, which yielded 16,380,270 SNPs for analysis after adjusting for factors such as age, sex, and genotyping batch [18]. Ethical approval and informed consent were provided for the above data, and the writing process followed the requirements of STROBE-MR, the reporting code for MR Studies. Data details are provided in Table 1.

**Table 1:** Bone mineral density (BMD) GWAS data summary

GWAS ID	Trait	Consortium/	Sample size	Trait	Reference
ebi-a-GCST00 5348	TB-BMD	GWAS meta- analysis	56 284	16 162 733	Medina et al., 2018 [19]
ieu-a-982	LS-BMD	GEFOS	28 498	10 582 867	Zheng et al., 2015 [20]
ieu-a-980	FN-BMD	GEFOS	32 735	10 586 900	Zheng et al., 2015 [20]
ieu-a-977	FA-BMD	GEFOS	8 143	9 955 366	Surakka et al., 2020 [21]
ebi-a-GCST00 6979	Heel-BMD	GEFOS	426 824	13 705 641	Morris et al., 2019 [22]

### Selection of IVs

The choice of IVs should conform to the above three assumptions. The SNPs (*P* < 5 × 10^− 8^) that were closely associated with BMD at the five sites were screened from the corresponding databases (Assumption 1). Second, SNPs pairwise correlation coefficients in linkage disequilibrium (LD) must satisfy r^2^ < 0.001 and kb = 10,000 to be considered independent (Assumption 2). In order to ensure that gene variants independently of potential confounders (Assumption 3), using PhenoScanner (http://www.phenoscanner.medschl.cam.ac.uk/) to screen and remove possible confounding factors. Known confounders that may be associated with the outcome (scoliosis) include vitamin D [23], age at menarche [24], body weight [25], BMI [26, 27], leptin [28], estrogen [29], melatonin [30], etc. The strength of instrumental variables was estimated using the F-statistic, which was calculated as F = (R^2^/k)/([1 − R2]/[n − k − 1]). R^2^ represents the proportion of variance explained by the exposed SNP tools, k represents the number of tools, and N represents the exposed sample size [31, 32]. F-statistics of less than 10 were considered to indicate weak instrumental variable bias and were excluded [33]. These SNPs were then used as IVs to assess causality between exposure and outcome in the MR Analysis.

### MR analysis

In this two-sample MR Design, we used the following five methods to analyze the causal association between BMD of different body parts and scoliosis separately [34–36]. These methods include inverse variance weighting (IVW), weighted median (WM), simple median (SM), weighted median estimator (WME) and MR-Egger regression. Among them, IVW calculates the odds ratio (OR) as the primary method, which is typical and routine in MR, and the slope of the weighted regression of the outcome effect on the exposure effect represents the outcome estimate (with an intercept restricted to zero). In addition, the other four methods were used as tests for the robustness of the primary outcome. Heterogeneity of individual estimates of genetic variation was assessed using Cochran’s Q test. If Cochran’s Q *P* > 0.05 and there was no evidence of heterogeneity, the fixed effect IVW method was used. If there was significant heterogeneity (*P* < 0.05 by Cochran’s Q test), the random effects IVW method was used [37–39].

### Sensitivity analysis

To ensure that IVs was independent of outcomes other than exposure, we used different approaches to exclude potential effects. Firstly, Pleiotropy residual sum and outlier (MR-PRESSO) was applied to test and calibrate the outliers of horizontal pleiotropy, and the outliers in IVs were removed [34]. We used MR-Egger regression to account for horizontal pleiotropy, with a *P* value of more than 0.05 for the intercept indicating the absence of horizontal pleiotropy [40]. Horizontal pleiotropy was tested by drawing a funnel plot. If the funnel plot shows a symmetric shape, this usually means that there is no obvious pleiotropy.

Leave-one-out sensitivity test is to observe whether the results will change significantly after removing a specific SNP. If the results remain relatively stable after removing any SNP one by one, it indicates that the overall error line will change within a small range after removing any SNP. Specifically, each SNP was removed one by one, and then the meta-effects of the remaining SNPs were calculated, in which the influence of each SNP was separately evaluated by IVW analysis and represented by forest plot.

### Analysis software

Statistical analysis of all data was performed using R Studio (version 4.3.1), two-sample MR (version 0.5.7) and MR-PRESSO (version 1.0) software packages [41]. Results are presented as odds ratios (ORs) with 95%confidence intervals (95% CI), and if *p* < 0.05 was considered statistically significant.

## Results

Overall, we obtained SNPs that met the three assumptions of MR Analysis and could be used for MR Analysis (Supplementary Table 1). In the heterogeneity test, all IVs did not show significant heterogeneity (*p* > 0.05), so a fixed effects model was used for the five-item MR Analysis when calculating the IVW.

IVW results were TB-BMD (OR = 0.83, 95%CI: 0.66–1.55, *P* = 0.13); LS-BMD (OR = 0.72, 95%CI: 0.52–0.99, *P* = 0.04) FN-BMD (OR = 0.74, 95%CI: 0.50–1.09, *P* = 0.13); FA-BMD (OR = 0.95,95%CI: 0.70–1.28, *P* = 0.75); HE-BMD (OR = 0.91, 95%CI: 0.77–1.08, *P* = 0.29). The complete results of the five MR Analyses are shown in Table 2. MR Study found a negative causal relationship between lumbar BMD and scoliosis (LS-BMD IVW OR = 0.72, 95%CI: 0.52–0.99, *P* = 0.04). There was no causal association between BMD at the remaining sites and scoliosis.

**Table 2:** Results of mendelian randomization analysis

		exposures				
		TB-BMD	LS-BMD	FN-BMD	FA-BMD	HE-BMD
SNPs, n		**63**	**18**	**14**	**11**	**419**
**IVW**	OR	0.83	**0.72**	0.74	0.95	0.91
	95%CI	0.66–1.55	**0.52–0.99**	0.50–1.09	0.70–1.28	0.77–1.08
	*P* value	0.13	**0.04**	0.13	0.75	0.29
Weighted median	OR	0.76	**0.57**	0.78	1.22	1.01
	95%CI	0.54–1.07	**0.36–0.90**	0.47–1.31	0.82–1.83	0.76–1.37
	*P* value	0.12	**0.01**	0.36	0.32	0.90
Weighted mode	OR	0.60	0.52	0.78	1.26	1.11
	95%CI	0.33–1.10	0.23–1.20	0.37–1.67	0.80–1.96	0.81–1.51
	*P* value	0.10	0.14	0.53	0.33	0.51
MR Egger	OR	1.43	1.17	2.02	1.22	1.06
	95%CI	0.80–2.58	0.30–4.56	0.29-14.00	0.51–2.91	0.80–1.42
	*P* value	0.24	0.82	0.48	0.66	0.65
Simple mode	OR	0.57	0.54	0.78	0.95	0.74
	95%CI	0.26–1.25	0.22–1.33	0.34–1.73	0.40–2.20	0.38–1.46
	*P* value	0.17	0.20	0.54	0.90	0.39

The boldface values represent the number of SNPS, the results of the main research methods, and the direct causal relationship (P < 0.05)

**Fig. 2:**
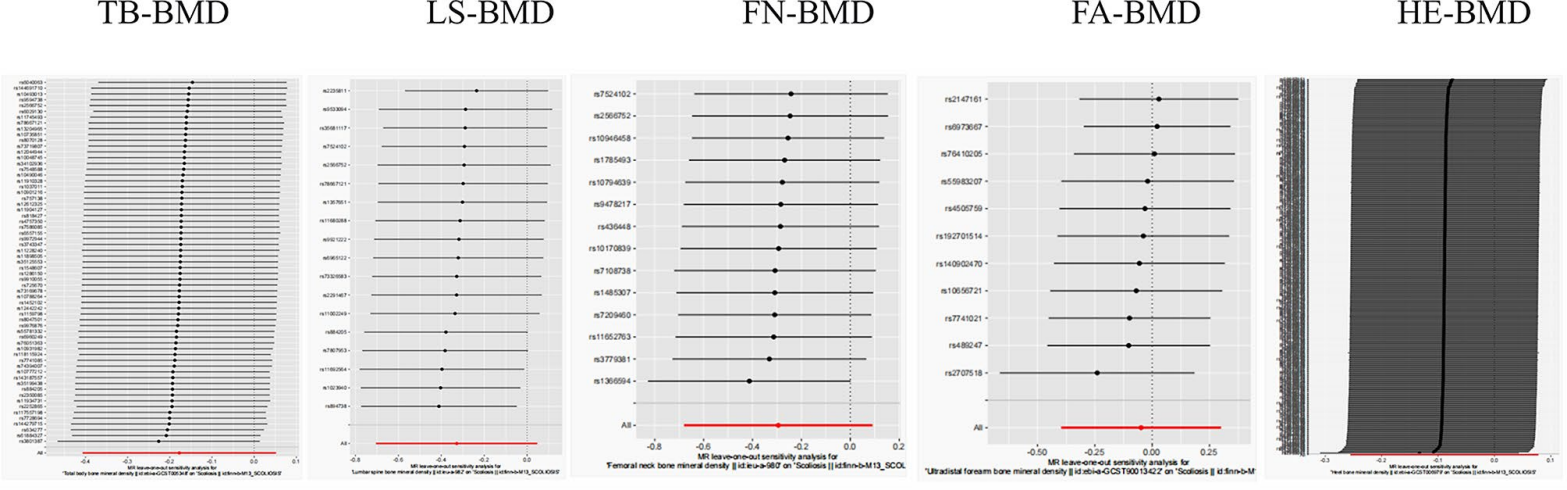
Analysis results of leave-one method

In the five MR Analyses performed, the *P* value for the intercept in the MR-Egger regression was higher than 0.05 (Table 2). No outliers were found by MR-PRESSO test. MR-Egger intercept and MR-PRESSO analysis showed that there was no pleiotropy of the above exposures on scoliosis, and there was no heterogeneity between them. In addition, the results of leave-one-out analyses performed and plotted show that no single SNP had an effect on the overall causal estimate (Fig. 2).

**Fig. 3:**
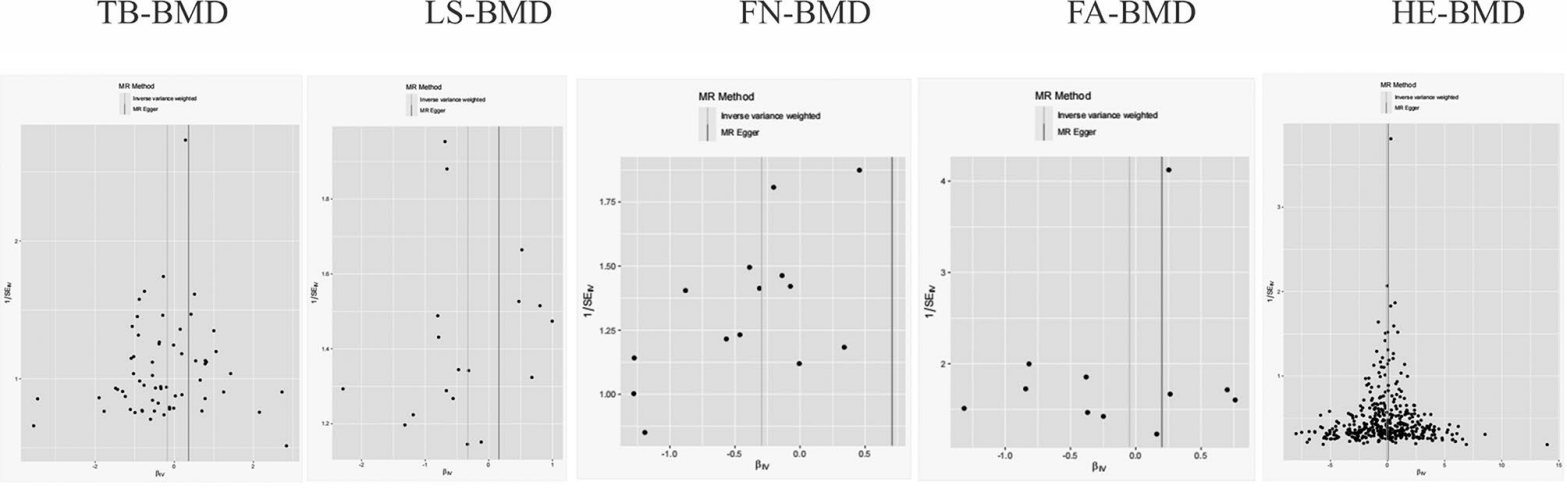
Funnel plot of causality between BMD and scoliosis

**Fig. 4:**
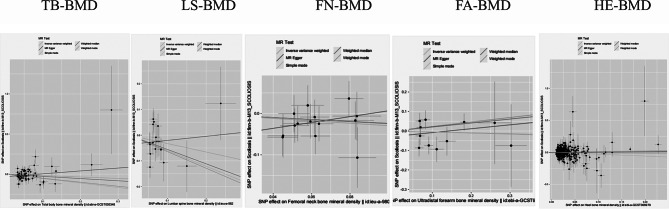
Scatter plot of causality between BMD and scoliosis

To further demonstrate the credibility of our findings, we plotted funnel plots and scatter plots for visual assessment of horizontal pleiotropy and heterogeneity. The distribution of causal effects shown in the funnel plot was basically symmetric and no obvious bias was observed (Fig. 3), and we found possible outliers in the IV of FN-BMD and FA-BMD in the scatter plot (Fig. 4). However, MR-PRESSO analysis showed no significant outliers (global test *P* > 0.05). Thus, the relationship between BMD and scoliosis provides insufficient support for horizontal pleiotropy. Reverse MR Analysis showed no causal relationship between scoliosis and BMD at the five sites (Supplementary Table 2).

## Discussion

So far, A variety of imaging methods have been used to measure BMD, such as dual-energy X-ray absorptiometry (DXA), quantitative ultrasound system (QUS), computed tomography, and quality control techniques (QCT) [42]. Many studies [43–46] have demonstrated the fact that BMD is reduced in patients with scoliosis by the above methods, but these studies have not proved whether there is a causal association between BMD at different locations and the presence or absence of scoliosis.

To the best of our knowledge, this is the first study to investigate the causal association between bone mineral density at different locations and scoliosis using two-sample MR Analysis. Studies have shown that lower lumbar BMD is associated with a significantly increased risk of scoliosis in a European population. BMD at other locations was not causally associated with scoliosis.

Cheng et al. [47] demonstrated that low LS-BMD may play an important role in the etiology and pathogenesis of scoliosis through bone histological studies, and low BMD is caused by metabolic disorders leading to a reduction in the number of osteoclasts in the trabecular compartment. Through DNA analysis of 198 girls diagnosed with AIS, Suh et al. [48] found that vitamin D receptor (VDR) BsmI polymorphism was associated with LS-BMD in AIS girls, and low bone mass may affect abnormal spinal growth patterns through VDR gene BsmI locus polymorphism.

The above studies have proved that LS-BMD plays an important role in scoliosis through various ways from different perspectives, but they have not revealed the direct causal relationship between the two determined by genes. Our results have proved the above conclusions, and our results are not affected by confounding factors, so the results are more reliable.

Handa et al. [49] found that in a longitudinal observation study in mice, the growth plate was thickened and osteoblasts were reduced over time, suggesting that impaired endochondral ossification was the cause of scoliosis. Reduction of bone mineral density and degradation of bone microstructure were also observed. This suggests that defects in endochondral ossification may impair growth, leading to scoliosis and decreased BMD. Therefore, more studies are needed in the future.

Our study has several strengths. Firstly, we applied the MR Method for the first time to investigate the causal relationship between BMD at different bone sites and scoliosis, avoiding potential confounding factors and reverse causality. Second, our data were derived from the GWAS, FinnGen, and the GEFOS consortium summary data, and the results were consistent across different datasets, ensuring the reliability of our findings.

However, this study still has some potential limitations. First, the database used in this study was based on populations of European ancestry, and it is unclear whether the results would apply to populations of non-European ancestry. Second, BMD and prevalence of scoliosis vary by age and sex, but this study was analyzed based on data from GWAS pooled levels, which does not allow subgroups to assess effects according to different age and sex. Third, the results of this study showed a significant causal relationship between LS-BMD and scoliosis, but not TB-BMD, FN-BMD, HE-BMD or FA-BMD. Further MR Studies with larger sample sizes or randomized controlled trials are needed to confirm these findings. Finally, Unobserved pleiotropy is a major limitation of MR Studies, which may influence conclusions that assess the association between genetically predicted BMD and scoliosis risk.

## Conclusions

In conclusion, this bi-directional two-sample MR Study found a causal relationship between LS-BMD and scoliosis, but found no evidence of a causal relationship between BMD at other sites and scoliosis. The lower the LS-BMD level, the higher the risk of scoliosis.

## Electronic supplementary material

Below is the link to the electronic supplementary material.

Supplementary Material 1

Supplementary Material 2

## Data Availability

No datasets were generated or analysed during the current study.

## Abbreviations

BMD: Bone mineral density
DXA: Dual energy X-ray absorptiometry
QUS: Quantitative ultrasonography
CT: Computerized tomography
GWAS: Genome Wide Association Study
MR: Mendelian randomization
SNPs: Single nucleotide polymorphisms
IVs: Instrumental variables
TB: Total body
LS: Lumbar spine
FN: Femoral neck
HE: Heels
FA: Forearm
IVW: Inverse variance weighting
WM: Weighted median
SM: Simple median
WME: Weighted median estimator
